# Sonographic landmarks in hamstring muscles

**DOI:** 10.1007/s00256-019-03208-x

**Published:** 2019-04-17

**Authors:** Ramon Balius, Carles Pedret, Iñigo Iriarte, Rubén Sáiz, Luis Cerezal

**Affiliations:** 1 Consell Català de l’Esport, Generalitat de Catalunya, Barcelona, Spain; 2 Sports Medicine and Imaging Department, Clínica Diagonal, Barcelona, Spain; 3 Clínica Mapfre de Medicina del Tenis, Barcelona, Spain; 4 Department of Rehabilitation, Clinica Ars, Bilbao, Spain; 5 Department of Radiology, Diagnóstico Médico Cantabria (DMC), Santander, Cantabria Spain

**Keywords:** Hamstring muscles, Ultrasound, Sonographic study, Tuning fork, Sonoanatomy

## Abstract

The ultrasound examination of hamstrings inspires respect due to the connective complexity of their structures, particularly for sonographers who are not used to this kind of study. Therefore, it is important to know the specific ultrasound reference points that facilitate the location of the hamstring structures, dividing them into four areas of interest: (a) tendinous origin of the hamstring, (b) the proximal half, (c) distal and medial half, and (d) distal and lateral half. The origin of the hamstrings is found at the level of the ischial tuberosity. Here, the connective structures under study are the common tendon and the semimembranosus tendon, together with the muscle fibers more proximal to the semitendinosus, which can also be assessed through ultrasound locating the ischial tuberosity. The proximal half of the thigh consists of a characteristic structure made up by the common tendon, the sciatic nerve and the semimembranosus tendon, enabling to define the biceps femoris and the semitendinosus, respectively. To identify the distal and medial section, the volumetric relationship between the ST and SM muscle masses is used, where it is also possible to identify the three muscles in the knee that make up the pes anserine. To identify the distal and lateral sections, the sciatic nerve pathway is followed until identifying both heads of the biceps femoris. These four areas of interest, with their specific landmarks, show a tuning fork that enables the comprehensive study of hamstrings through ultrasound.

**Electronic supplementary material:**

The online version of this article (10.1007/s00256-019-03208-x) contains supplementary material, which is available to authorized users.

## Introduction

Ultrasound examination of the hamstring muscles involves a demanding technique and an in-depth anatomical knowledge of the area. Imaging specialists dealing with musculoskeletal ultrasound (MSUS) are already familiar with normal anatomy and US anatomy, but the volume and presence of large intramuscular connective expansions, as well as the fact that none of these muscles have a uniform architecture, gives the hamstrings a certain structural complexity. This results in a more complex ultrasound analysis than in any other anatomical region, especially in the case of inexperienced sonographers who are at the beginning of their learning curve.

## Hamstring anatomy

**Fig. 1:**
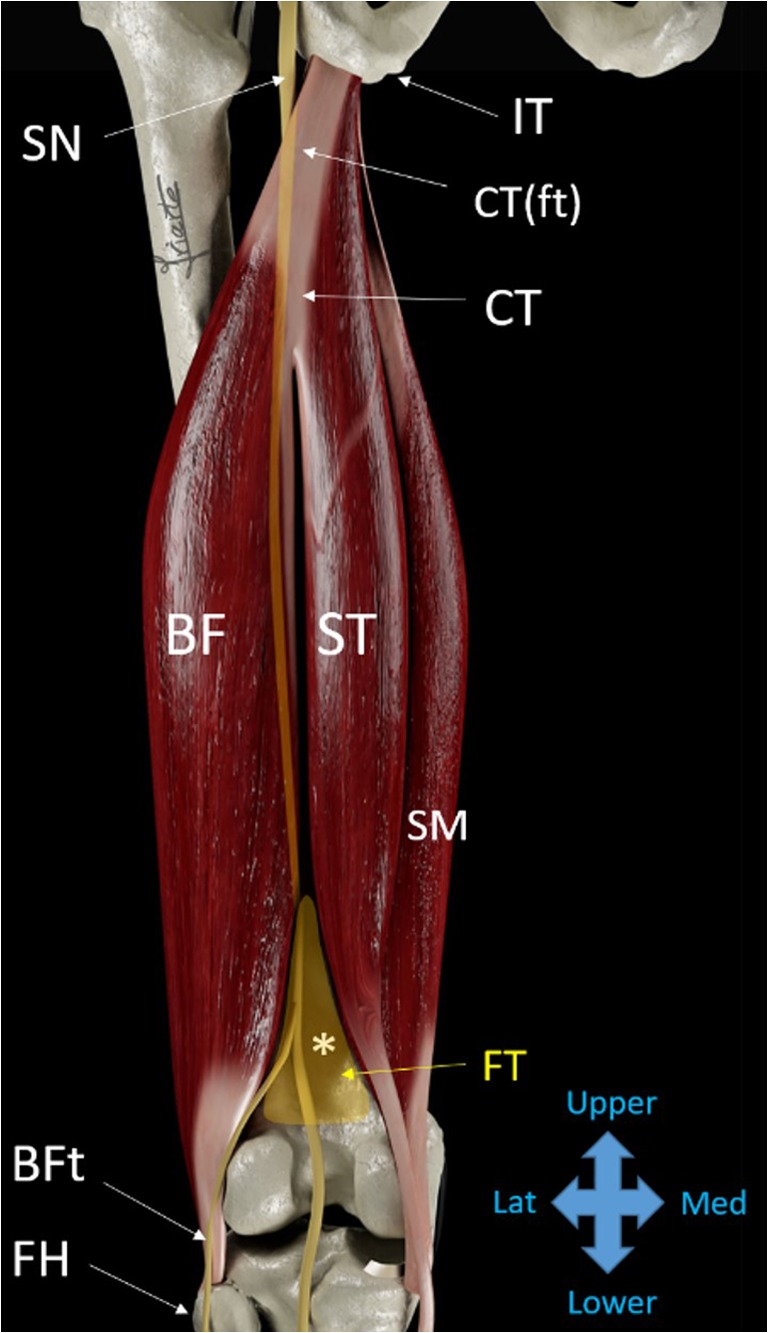
Diagram of the hamstring muscles. *BF* biceps femoris muscle, *ST* semitendinosus muscle, *SM* semimembranosus muscle, *CT* common tendon, *CT(ft)* free-tendon part of the common tendon. *SN* sciatic nerve, *IT* ischial tuberosity, *FH* fibular head, *FT* fat tissue in the space between the BF and ST/SM muscles, (*) Popliteal vessels and nerve are interposed between the BF and ST/SM muscles

The hamstrings consist of the semimembranosus (SM), semitendinosus (ST), and biceps femoris (BF) muscles. The latter has both a long and a short head (Fig. 1). They all arise from the posterior, proximal, and lateral sides of the ischial tuberosity [1, 2], reaching the leg through aponeurotic extensions and tendons. Thus, the insertion of the semitendinosus and semimembranosus is on the medial side of the tibia; whereas the biceps femoris attaches to the head of the fibula. While the biceps femoris muscle is superficial and lateral, the semitendinosus is superficial and medial. Together, they make up the entire muscle mass of the proximal part of the hamstrings. The muscle mass of the semimembranosus has a more distal origin, increasing toward the distal and medial parts of the thigh. The short head of the biceps femoris also originates from the distal and lateral part of the thigh, along the *linea aspera* of the femoral diaphysis and the ventral part of the distal tendon of the long head [1]. More caudally, both heads of the biceps femoris meet in a common distal tendon reaching the head of the fibula. Deep into the hamstrings, the large muscle mass of the adductor magnus is found (Fig. 1).

**Fig. 2:**
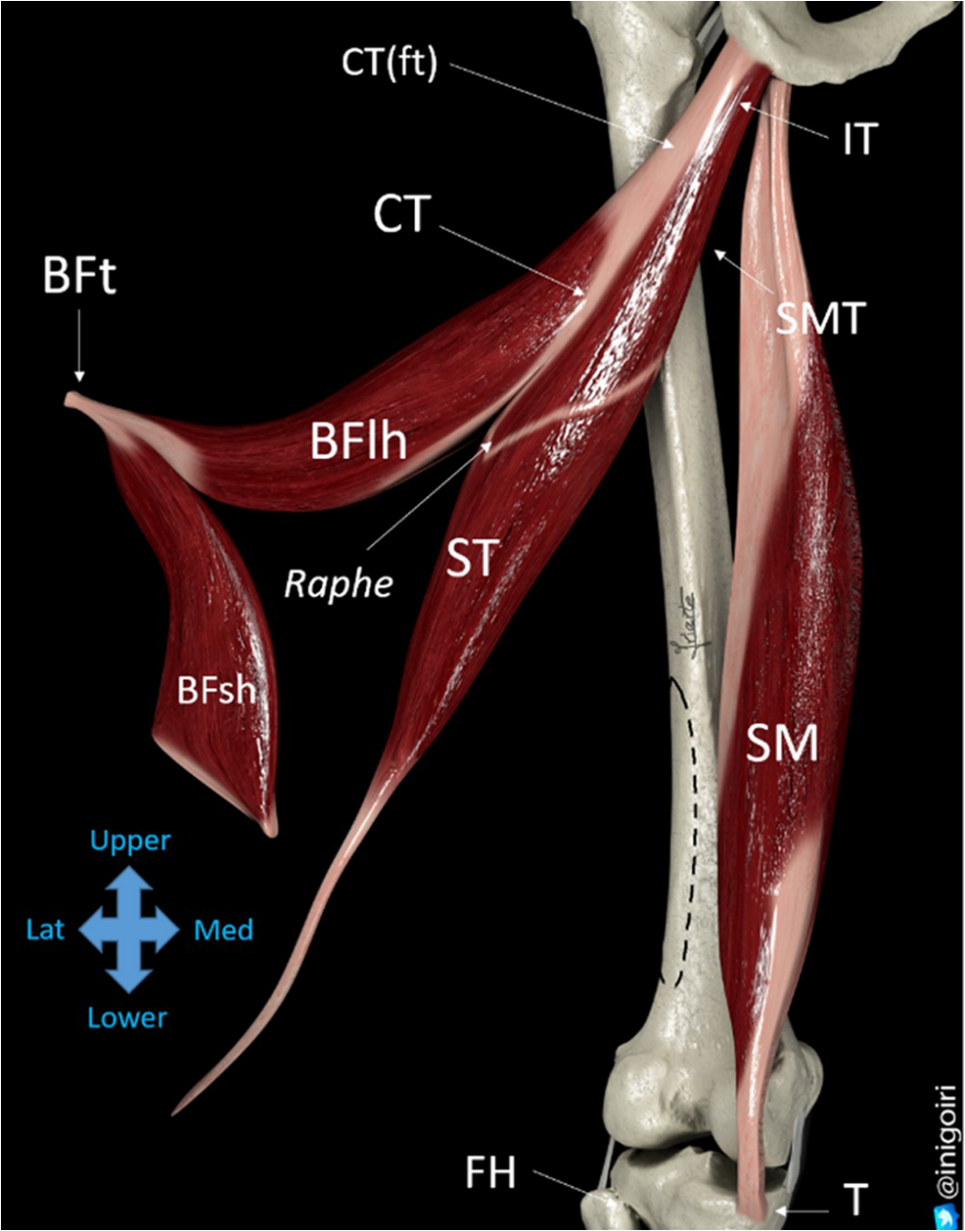
Diagram of expanded hamstring muscles. *BFlh* long head of the biceps femoris muscle, *BFsh* short head of the biceps femoris muscle, *ST* semitendinosus muscle, *SM* semimembranosus muscle, *SMT* semimembranosus tendon, *CT* common tendon, *CT(ft)* free-tendon part of the common tendon. The *dotted line* on the femoral diaphysis marks the *linea aspera*, the origin of the BFsh. *IT* ischial tuberosity, *T* medial side of the tibia, *BFt* distal BF tendon

The long head of the biceps femoris has a tendon that is 6–9 cm long [2, 3] and a free part of the musculotendinous junction, called free tendon, with a variable length of about 5 ± 3.4 cm [2, 4]. Distally, the long head has shorter fascicles and a greater pennation angle [5] (Fig. 2).

The semitendinosus has a connecting ridge roughly located in the proximal quarter of the muscle belly, separating the muscle into two independent units with different innervation from the tibial component of the sciatic nerve [3]; for this reason, it may be considered a digastric muscle [2]. It is the only hamstring with muscle fibers directly reaching the ischial tuberosity [1, 3].

The proximal aponeurosis of the biceps femoris and semitendinosus forms a conjoint tendon [2], where one of the most common muscle injuries in the world of sports occurs [6–8]. In this case, it is very important to identify whether it is a free-tendon or purely myotendinous injury [9].

The first muscle fibers of the semimembranosus originate at 30% along the entire length of the muscle [3]. Furthermore, it has a powerful tendon measuring 9.4 ± 2.6 cm [2] that runs distal and ventral to (i.e., deep to) the semitendinosus.

The sciatic nerve passes through the biceps femoris ventrally from the lateral to the medial sides and from cephalad to caudal. Located laterally to the ischial tuberosity, it then enters the subgluteal space, very close to the origin of the semimembranosus tendon [10]. Therefore, in the proximal thigh, the sciatic nerve is located ventrally and in contact with the biceps femoris virtually all the way, whereas in the distal half, it is medial to the short head and posterior to the long head [2, 11].

The hamstrings split at the proximal section of the popliteal fossa. The semimembranosus and semitendinosus attach to the medial side of the knee, while both heads of the biceps femoris attach to the lateral side. In this area, a lot of fat tissue fills out the space between muscles and tendons [12].

On the medial side, the distal tendon of the semimembranosus acts as the main stabilizer of the posteromedial complex of the knee. It has five main extensions, although up to eight have been described [13]: direct portion (main insertion), capsular portion, extension that joins the oblique popliteal ligament, anterior (tibial or reflex) portion, and distal (popliteal) portion. The tendon of the semimembranosus is an important dynamic posteromedial stabilizer of the knee, primarily during flexion (lax posterior oblique ligament) and internal rotation of the knee [14, 15]. On the lateral side, the short and long heads of the biceps femoris form one single tendon that attaches to the head of the fibula, encompassing the distal insertion of the lateral collateral ligament.

## Ultrasound examination

Classically, ultrasound examination of the hamstrings has preferably used osseous landmarks [5, 16–19] or prior knowledge of topographic muscle anatomy [12, 20, 21]. The area of interest of these articles focuses especially on the insertion of the hamstrings [16–18], using manual or US examination to locate the osseous profile of the ischial tuberosity. Assessment of the proximal half of the hamstrings is aided by locating muscle masses in the short axis. In this regard, Jacobson [20] recommends starting the examination on the medial side, identifying the triangular section of the SM and moving from there along the short axis in a lateral direction, identifying the sections of the ST and the BF. Bianchi and Martinoli [12], on the other hand, also suggest identifying the section of the SM in a short-axis plane, but moving distally before sweeping the transducer laterally to find the small round section of the ST and, more laterally, the section of the BF. Habelfehlner [21] recommends directly identifying the small section of the semitendinosus at the level of the popliteal fossa. The lateral distal half, corresponding to the biceps femoris, is examined using the head of the fibula as a landmark [5] or the anatomical “split” with respect to the ST and SM at the level of the popliteal fossa [12].

### Examination position and probe

The patient is placed in the prone position, with their feet hanging off the edge of the table. Multifrequency 6–10-MHz probes are usually used but lower frequencies are recommended when examining obese or very muscular patients, particularly in the area of origin of the ischial tuberosity.

### Areas of study

**Fig. 3:**
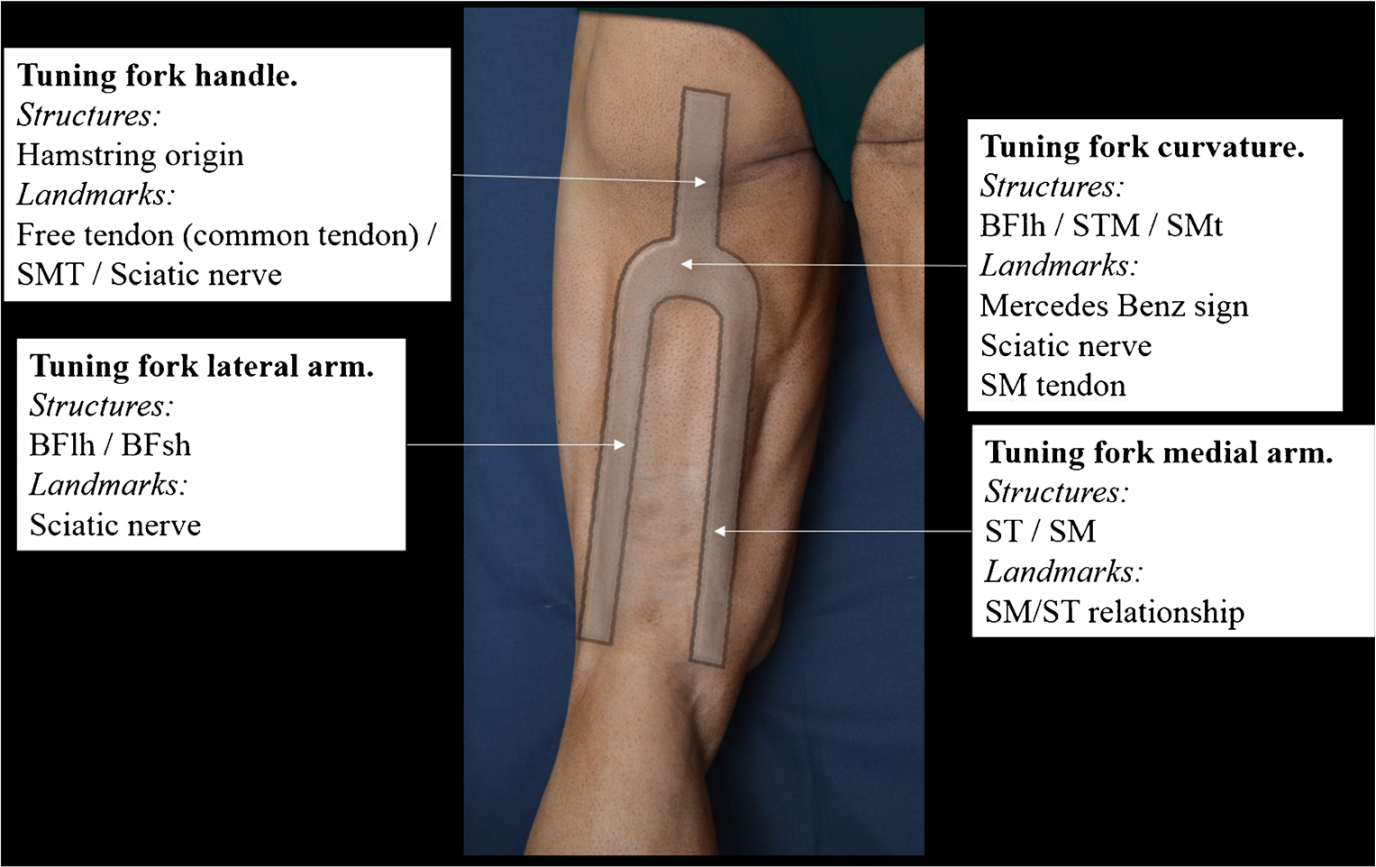
The hamstrings form an inverted tuning fork with four areas of study: the handle, the curve, and two arms. Each of these areas contains interesting anatomical structures and specific ultrasound landmarks that facilitate a systematic study of the hamstrings. *BFlh* long head of the biceps femoris muscle, *BFsh* short head of the biceps femoris muscle, *ST* semitendinosus muscle, *SM* semimembranosus muscle, *SMT* semimembranosus tendon

The sonographic study of the hamstrings looks like an inverted tuning fork, marking four areas of interest with specific landmarks (Fig. 3).

- The handle of the tuning fork leads to the origin of the hamstrings in the ischial tuberosity. It consists of the common tendon (free tendon at this level) and the semimembranosus tendon.
- The curved area of the tuning fork corresponds to the proximal half of the hamstrings. It mainly consists of the biceps femoris, ST muscles and SM tendon. The landmarks at this level are the sciatic nerve and the semimembranosus tendon.
- The medial arm of the tuning fork consists of the SM and ST muscle masses. To study this area, the volumetric relationship between the ST and SM muscle masses is followed along a short axis, proximal to distal. At a proximal level, the muscle mass that prevails is that of ST. As it decreases, the relationship between the ST and SM muscle masses switches so that ST is much larger than SM at a proximal level while SM is the largest at a distal level.
- Finally, the two heads of BF make up the lateral arm of the tuning fork. To study this area, it is enough to follow the short axis of the sciatic nerve pathway distally.

### Examination technique

It is recommendable to start the sonographic technique by locating the origin of the hamstrings (handle of the tuning fork) to then progress with the examination distally to the remaining areas. A second option is to start with the proximal-mid thigh (curved area of the tuning fork) and from there, progress with the analysis in a short axis towards the origin before studying the distal thigh, both medial and lateral (arms of the tuning fork). This second option is reserved for patients in whom it is difficult to locate the ischial tuberosity ultrasonographically (for example obese or extremely muscular patients).

### Ultrasound study of the origin of hamstrings in the ischial tuberosity (Fig. 4)

Although MRI has a better resolution at this level [22], ultrasound can also be used to assess the origin of the hamstrings in the ischial tuberosity and their topographical relationship with the sciatic nerve. In the ischial tuberosity, the origin of the hamstrings is close to the sciatic nerve, which is located more laterally. For this, we need a surface landmark, e.g., just caudal to the gluteal cleft. It is in this place that we place the transducer first in order to find a strong hyperechoic line with a posterior acoustic shadow consistent with the ischial tuberosity. In most cases, we can see the ST muscle fibers there, because this muscle reaches the ischial tuberosity directly. As the probe is moved distally, the muscle section of the BF can be seen to widen like a triangle shape, while the ST also increases in size. The origin of the BF/ST common tendon, lateral to the ischial tuberosity and with a more hyperechoic and superficial appearance, can be seen, as can the SM tendon (also echoic), which is more lateral, deep, and close to the sciatic nerve section. By modifying the angle of the ultrasound beam of the probe, a certain degree of anisotropy can be created, which helps to differentiate the SM from the conjoin tendon.

This can be very important for differentiating whether an injury is located in the conjoint tendon or the tendon of the SM and establishing ultrasound-guided or surgical treatment [16, 17, 23]. The sections of the common tendon and the SM tendon coincide with the handle of the tuning fork in a short axis.

### Ultrasound of the proximal-mid thigh (Fig. 5)

This area refers to the examination along the short axis at the junction of the proximal and middle thirds of the thigh. At this level, the section of the sciatic nerve can be identified easily as an oval or flat structure, arranged in a fascicle and surrounded by hyperechoic fat [24]. This structure is constant and easily visible because it has virtually no anisotropy. The sciatic nerve is found in the center of a distinctly hyperechoic, three-point star, similar to the iconic Mercedes Benz logo.

**Fig. 4:**
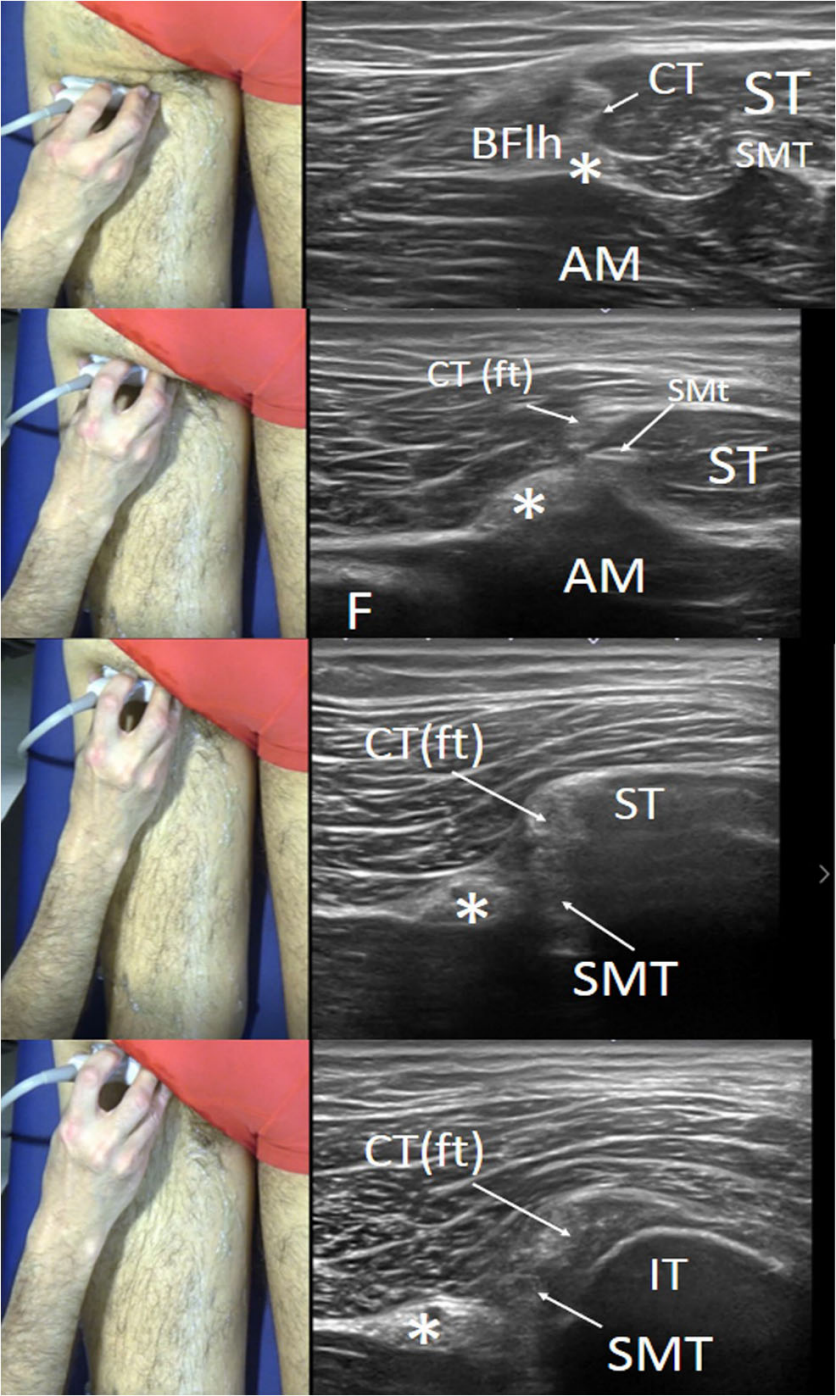
Series of short-axis ultrasound scans showing different views of the proximal part of the hamstrings (handle of the tuning fork). As the probe is moved in a proximal direction, the sections of the common tendon and the semimembranosus tendon can be seen approaching the sciatic nerve until a hyperechoic line is observed with a posterior acoustic shadow consistent with the ischial tuberosity. *BFlh* long head of the biceps femoris, *ST* semitendinosus muscle, *AM* adductor magnus muscle, *SMT* semimembranosus tendon, *CT* common tendon, *CT (ft)* free-tendon part of the common tendon, (*) sciatic nerve. The photographs on the left of the figure indicate probe positioning

The proximal point corresponds to the conjoint tendon with the (medial) semitendinosus muscle and (lateral) biceps femoris located on each side. At this level, the ST section is somewhat larger than the BF. If the transducer is moved to proximal, the size of the BF decreases and assumes a triangular shape that progressively disappears. From this view, and moving the probe in a proximal direction, the part belonging to the biceps femoris free tendon starts. The ST section remains visible as far as the ischial tuberosity. From the medial part of the sciatic nerve arises a hyperechoic line that corresponds to the membrane of the semimembranosus, which ends in the semimembranosus tendon (it is oval and smaller than the sciatic nerve), running virtually parallel to it. Ventral to these muscles, the adductor magnus is found.

The sciatic nerve runs through the thigh from the medial to lateral sides and from cephalad to caudal deep to the biceps femoris, whereas the tendon of the semimembranosus runs right deep to the semitendinosus muscle. Therefore, when the probe is placed longitudinally over the sciatic pathway, the biceps femoris can be seen above it, and when placed along the tendon of the semimembranosus, the semitendinosus muscle can be observed. The latter has one last ultrasound landmark to check: a characteristic thin connecting ridge (or raphe) in the muscle thickness which is constant and hyperechoic and can be seen when examining it in both the axial and the sagittal planes [12] (Fig. 6).

**Fig. 5:**
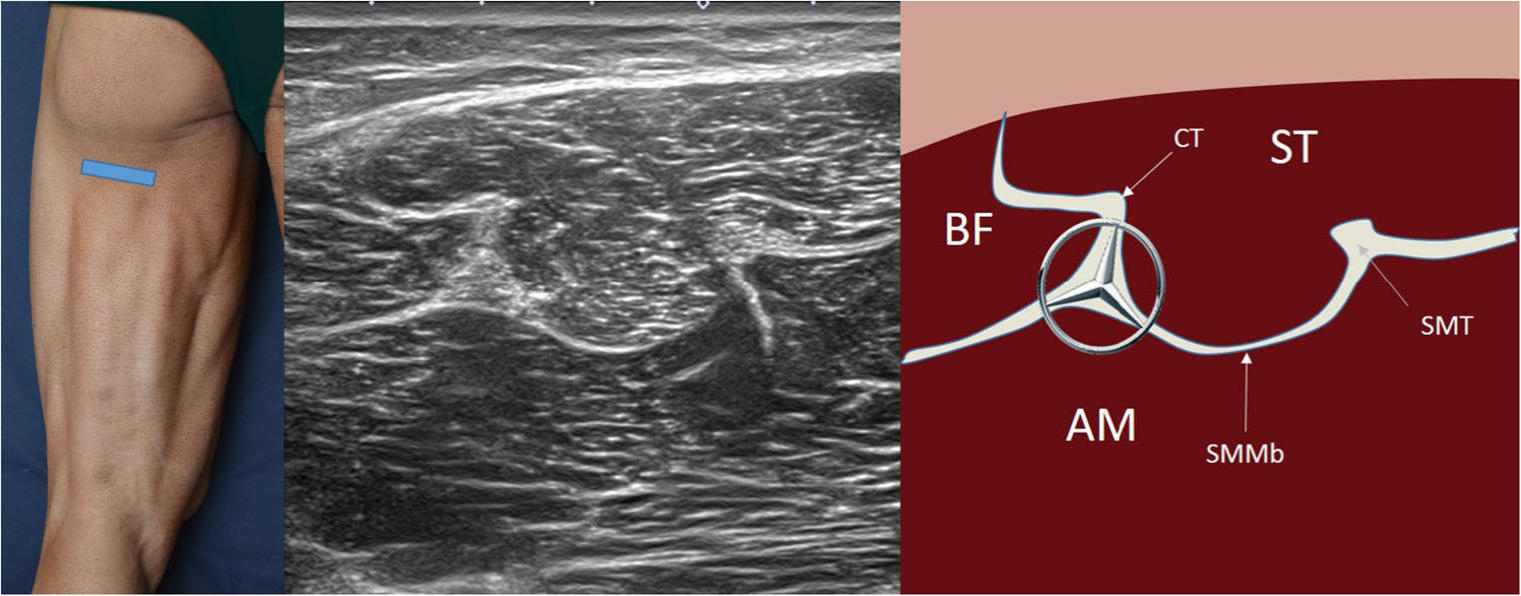
Short-axis ultrasound view of the proximal-mid third of the back thigh with comparative diagram. The section of the sciatic nerve can be seen like the main landmark (remember the iconic Mercedes Benz logo). *BF* biceps femoris muscle, *ST* semitendinosus muscle, *AM* adductor magnus muscle, *CT* common tendon, *SMMb* semimembranosus membrane, *SMT* semimembranosus tendon. The photograph on the left of the figure indicates probe positioning

### Ultrasound of the distal and medial thigh (Fig. 7)

From this first study point, the probe is moved distally along the medial arm of the tuning fork. For this purpose, the probe is moved medially (always in a short axis) so that the more medial edge of the semitendinosus can be seen, and then it is distally moved, locating the muscle fibers that are more proximal to the semimembranosus, which, at this level, have a semi-circular shape in the lateral concavity. This image should not be confused with that made by the connective ridge of the semitendinosus, which has a medial concavity. The ST/SM connection is followed distally and medially in a short axis to the thigh. As the probe moves distal, the semi-circle of the SM can be seen to increase in size, while the semitendinosus decreases.

**Fig. 6:**
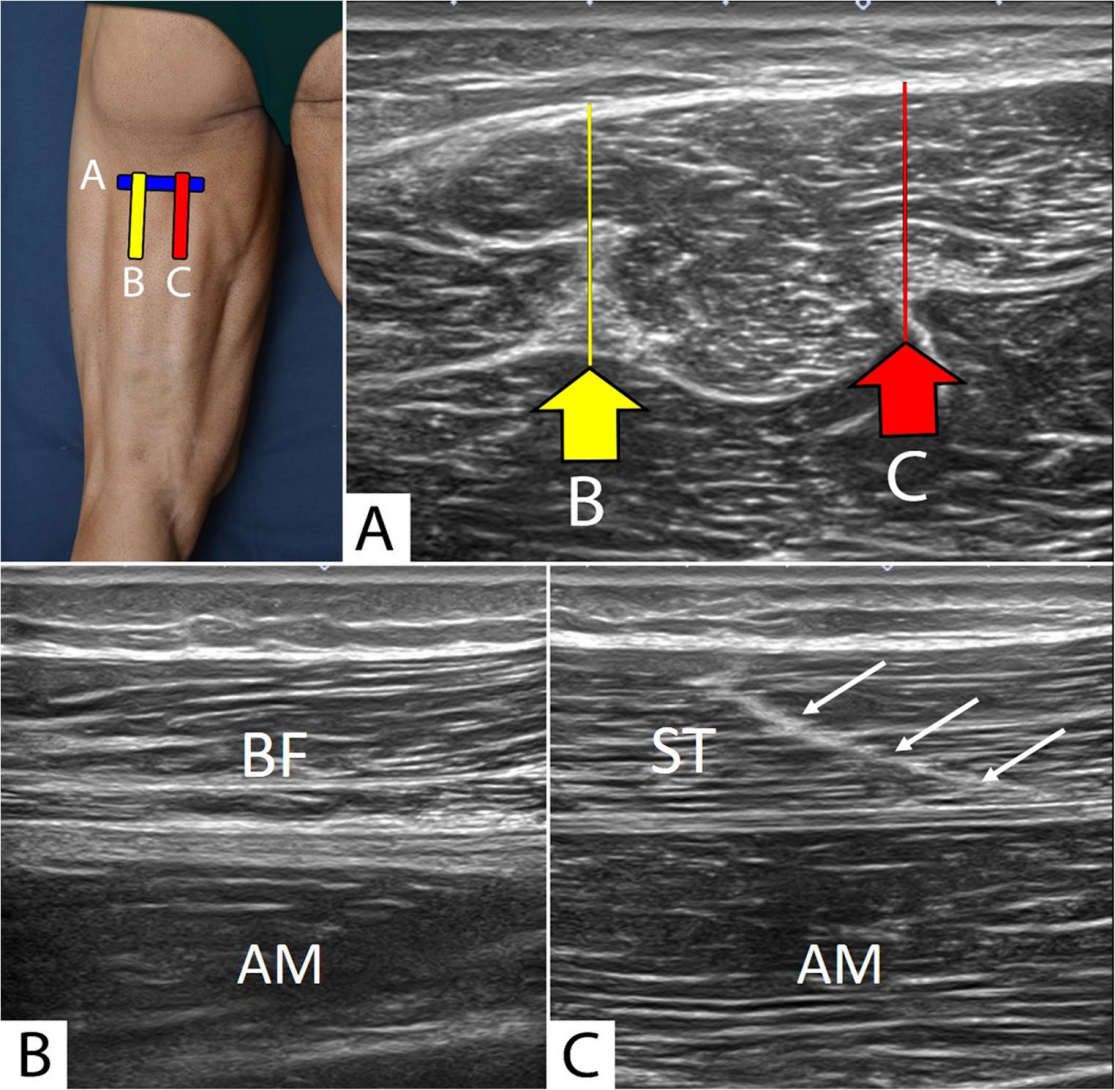
**a** Short-axis ultrasound view of the proximal third of the back thigh. **b** By placing the probe in the long axis over the sciatic nerve, the biceps femoris muscle can be located. **c** By placing the probe in the long axis over the semimembranosus tendon (C), the semitendinosus muscle with its raphe can be located. The adductor magnus is located ventral to these structures. *BF* biceps femoris muscle, *ST* semitendinosus muscle, *AM* adductor magnus muscle, *White arrows* raphe of the semitendinosus. The photograph in the top left of the figure indicates probe positioning

On reaching the distal and medial sections of the thigh, the semitendinosus assumes an oval shape, located superficially and laterally to the large mass of the semimembranosus, with a characteristic hyperechoic cordon structure consistent with the distal tendon of the ST. At this point, if the probe is moved medially, two circular sections can be found transversally in the inner thigh, one next to the other: the sartorius muscle, more anterior and with a thicker section, and the gracilis, immediately after the former and with a much smaller section. Therefore, the muscle circles that can be seen, from ventral to dorsal, corresponds to the sartorius muscle, the gracilis and the semitendinosus, the so-called “pes anserine muscles”. The entire muscle area located between the gracilis and the semitendinosus corresponds to the large mass of the semimembranosus, which has a rough structure due to its thick, diversely positioned fibers (Fig. 8).

**Fig. 7:**
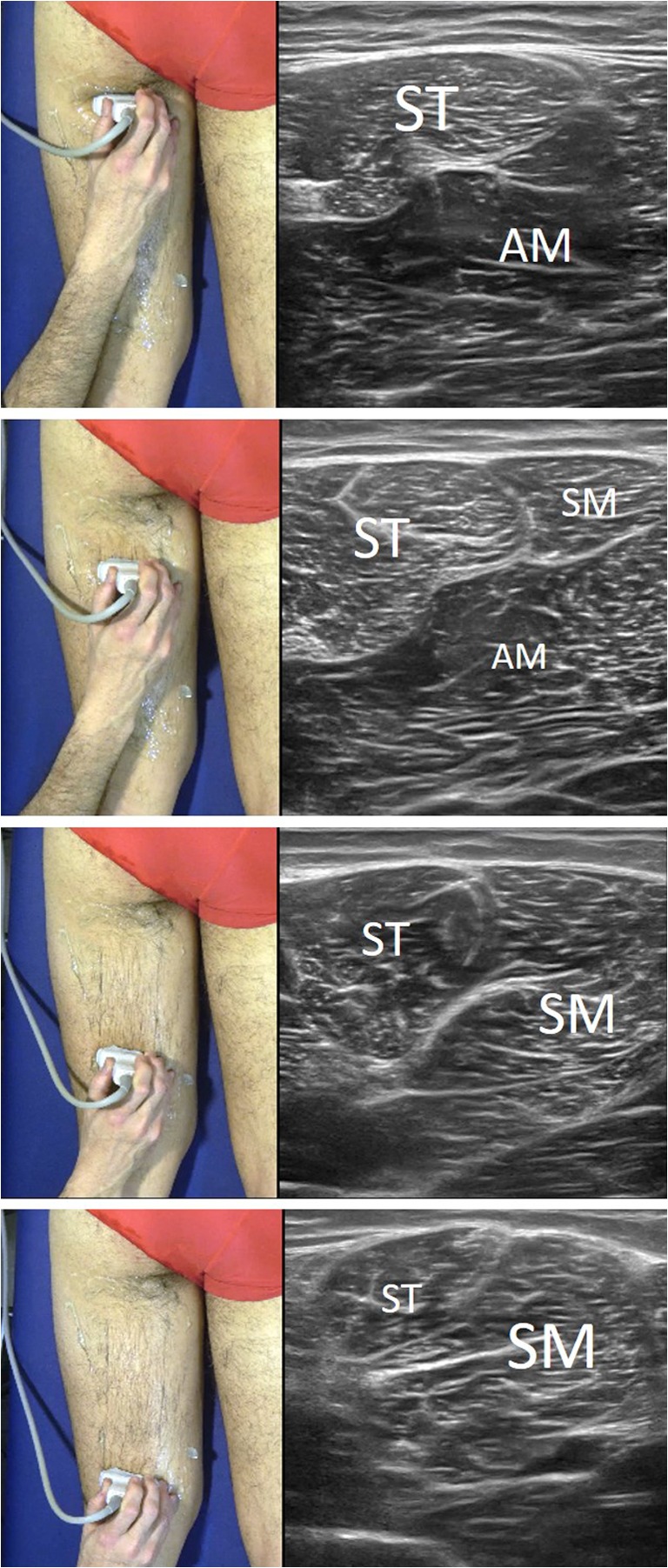
Series of short-axis ultrasound scans showing different views of the medial part of the hamstrings (medial arm of the tuning fork). As the probe is moved distally, the SM muscle mass can be seen to increase in size, while that of the ST decreases. The photographs on the left of the figure indicate probe positioning. (*ST* semitendinosus muscle, *SM* semimembranosus muscle AM: aductor magnus muscle)

### Ultrasound of the distal and lateral thigh (Fig. 9)

The probe is relocated in the proximal half. From here, it is moved to the distal and lateral sections along the lateral arm of the tuning fork, i.e., following the sciatic nerve in a caudal direction along a short axis. In the middle third of the back thigh, the biceps short head appears as a growing fusiform structure arising from the *linea aspera* of the femur, located between the long head and the lateral vastus muscle. This point is important because the short head of the biceps muscle belly is an important landmark to differentiate between proximal and distal hamstring injuries; this is routinely used in MRI and can also be used in ultrasound examination. Transversally, the short head has a similar shape to a quadrangle inserted in the hyperechoic profile of the femur. Right next to it, and medially to the short head, the long head (with a more triangular shape) is found. About 6 cm from the popliteal fold, it is possible to observe the bifurcation of the sciatic nerve into the common fibula nerve and the tibial nerve (Fig. 9).

**Fig. 8:**
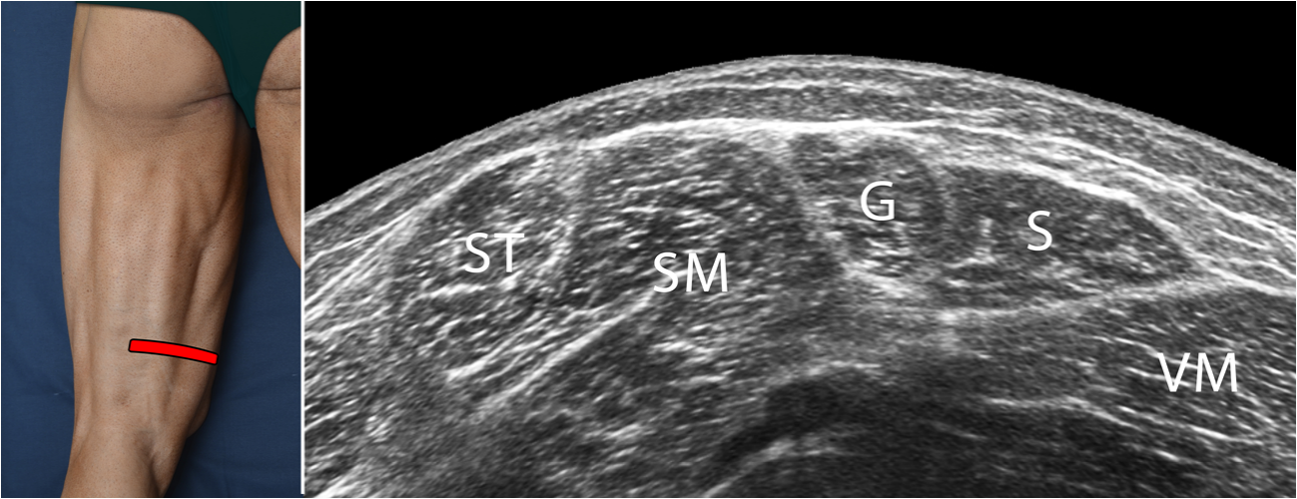
Panoramic ultrasound view of the distal section of the back thigh showing the pes anserine muscles that, from ventral to dorsal, correspond to the semitendinosus (ST), gracilis (G), and sartorius (S) muscles. The entire muscle area between G and ST is consistent with the large muscle mass of the semimembranosus (SM). *VM* vastus medialis muscle. The photograph on the left of the figure indicates probe positioning

**Fig. 9:**
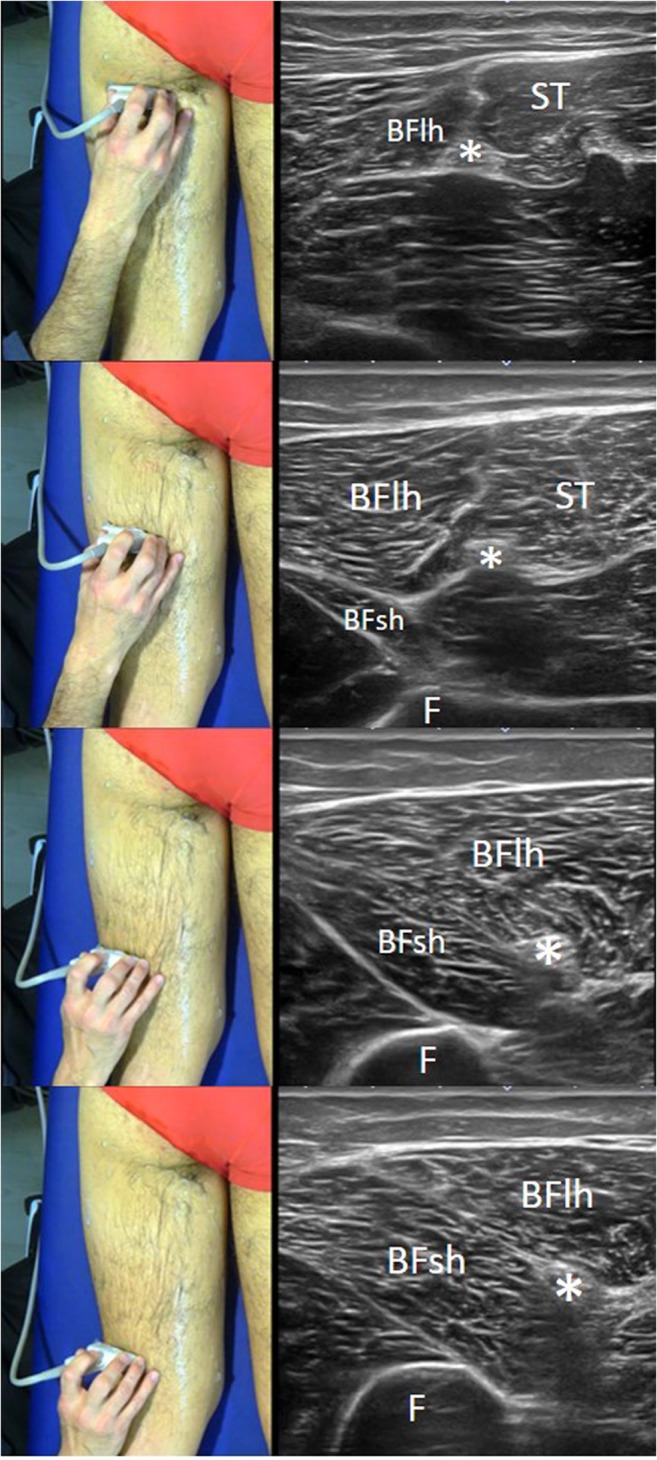
Series of short-axis ultrasound scans showing different views of the lateral part of the hamstrings (lateral arm of the tuning fork). As the probe is moved distally along the sciatic nerve pathway, the section of the short head of the biceps femoris muscle can be seen to appear. The photographs on the left of the figure indicate probe positioning. *BFlh* long head of the biceps femoris, *BFsh* short head of the biceps femoris, *ST* semitendinosus muscle, (*) sciatic nerve

## Discussion

We strongly believe that our article offers a novel approach because it analyzes ecographic landmarks that have not been bibliographically referenced until now and makes an attempt to group all of them globally. In this regard, we have reviewed the “classic” textbooks on MSUS. Some of them, such as the book by M. Van Holsbeeck [25] do not cover hamstring examination. In the book by J.A. Jacobson [20], in chapter 6 the author describes a system based on knowledge of the relationship between muscle masses. Our system is based on echographic landmarks that are often situated in the intramuscular structure of the hamstrings (e.g., SM tendon) or the sciatic nerve (e.g., its relationship with the BF). In the book by S. Bianchi and C. Martinoli [12], in chapter 13 the authors propose that the hamstrings should be studied in a short-axis plane and again basing the examination on relationships between muscle masses. These authors do use the sciatic nerve to locate the biceps femoris. With our system, the hamstrings can be assessed globally by relating all the structures with the aid of the proposed landmarks. We have also conducted a systematic review on PubMed, searching for publications where this new approach may already have been presented. A 15-year search was performed (2003–2018) using the following keywords: (“hamstring muscles”[MeSH Terms] OR (“hamstring”[All Fields] AND “muscles”[All Fields]) OR “hamstring muscles”[All Fields] OR “hamstring”[All Fields]) AND (“diagnostic imaging”[Subheading] OR (“diagnostic”[All Fields] AND “imaging”[All Fields]) OR “diagnostic imaging”[All Fields] OR “ultrasound”[All Fields] OR “ultrasonography”[MeSH Terms] OR “ultrasonography”[All Fields] OR “ultrasound”[All Fields] OR “ultrasonics”[MeSH Terms] OR “ultrasonics”[All Fields]) AND (“anatomy and histology”[Subheading] OR (“anatomy”[All Fields] AND “histology”[All Fields]) OR “anatomy and histology”[All Fields] OR “anatomy”[All Fields] OR “anatomy”[MeSH Terms]). In total, 109 papers were identified, only 13 of which included a description of the hamstrings. The eligibility criteria were that the study involved the use of US, thus excluding six papers. Finally, a total of seven studies met the selection criteria. Bengtzem et al. [16] assessed acute hamstring rupture by US. They started their assessment in the long axis. Their description is limited to cases of injury and they do not specify a regular system. The echographic landmark they use is the osseous profile of the ischial tuberosity. Burke et al. [17] do not specify a regular system for hamstring examination either, and they use a short-axis ultrasound approach in two hamstring-origin calcific tendinopathy barbotage procedures. Haberfehlner at al. [21, 26] performed “measurements of ST morphology using 3DUS. A 30 ± 40-s sequence of transverse US images (i.e., axial plane of the ST) was collected starting distally at the ST tendon (i.e., at the point that the tendon could be sufficiently visualized in the popliteal fossa) to the origin on the ischial tuberosity”. Kellis et al. [18] used ultrasound to validate the architectural properties of the hamstring muscles, performing a correlation of US findings with dissection on three cadavers. To standardize the US probe positions, the origin of the ST and BF is determined initially. In particular, the common proximal BF and ST tendon at the lateral aspect of the medial portion of the ischial tuberosity is identified by taking axial and longitudinal scans. The distal origin of the ST is identified as the point where the ST inserts into the gracilis tendon and subsequently into the fascia cruris. The distal origin of the BF is from the inferior margin of the fibular head. Palmer et al. [19] carried out a “reliability” study of “panoramic US” to explore whether it could be a “reliable technique for examining muscle size and quality of the hamstrings”. These authors performed a panoramic US imaging assessment of a cross-sectional area along the midline between the osseous landmarks of the greater trochanter and the “lateral joint line of the knee”. Tosovic et al. [5] carried out an US study of the long head of biceps femoris, using osseous landmarks. They identified the cranial area from the ischial tuberosity and the more caudal area from the head of the fibula.

In conclusion, based on bibliographic research, no publication has been found that reports a systematic US description of the hamstrings as in the approach presented here, where a systematic ultrasound examination has been described, dividing the hamstrings into four areas of interest based on constant, characteristic landmarks. This ultrasound methodology will allow sonographers to achieve a global and reproducible view of the hamstrings, avoiding any confusion of critical structures for an adequate image analysis (Video 1). We strongly believe that our article is a novel approach because it analyzes echographic landmarks that have not been bibliographically referenced until now and makes an attempt to group them all globally.


(MP4 162,032 kb)


## Abbreviations

BF: Biceps femoris muscle
ST: Semitendinosus muscle
SM: Semimembranosus muscle
CT: Common tendon
SN: Sciatic nerve
AM: Adductor magnus muscle
SMT: Semimembranosus tendon
G: Gracilis muscle
S: Sartorius muscle
BFlh: Long head of biceps femoris
BFsh: Short head of biceps femoris
QF: Quadratus femoris muscle
IT: Ischial tuberosity
